# Adalimumab in Japanese patients with active ulcers of pyoderma gangrenosum: Twenty‐six‐week phase 3 open‐label study

**DOI:** 10.1111/1346-8138.15533

**Published:** 2020-08-17

**Authors:** Kenshi Yamasaki, Keiichi Yamanaka, Yiwei Zhao, Shunsuke Iwano, Keiko Takei, Koji Suzuki, Toshiyuki Yamamoto

**Affiliations:** ^1^ Department of Dermatology Tohoku University Graduate School of Medicine Sendai Japan; ^2^ Department of Dermatology Graduate School of Medicine Mie University Tsu Japan; ^3^ AbbVie Inc. Cambridge Massachusetts USA; ^4^ AbbVie GK Tokyo Japan; ^5^ Department of Dermatology Fukushima Medical University School of Medicine Fukushima Japan

**Keywords:** adalimumab, Japanese patient, pyoderma gangrenosum, skin ulcer, tumor necrosis factor‐α

## Abstract

This phase 3 multicenter study, including 26‐week treatment and extension periods, evaluated the efficacy and safety of adalimumab in Japanese patients with active ulcers due to pyoderma gangrenosum. Patients received adalimumab 160 mg at week 0, 80 mg at week 2, and then 40 mg every week starting at week 4. Of the 22 enrolled patients, 12 (54.5%, *P* < 0.001) achieved the primary efficacy end‐point of pyoderma gangrenosum area reduction 100 (PGAR 100, defined as complete skin re‐epithelialization) for the target ulcer at week 26 assessed by digital planimetry. PGAR 100 response was observed as early as week 6 (13.6%) and continued to increase over time. The mean percent change from baseline in target ulcer area was −31.8% at week 6 and −63.8% at week 26. A Physician’s Global Assessment score of 0 (PGA 0, all ulcers completely clear) was achieved by two patients (9.1%) at week 6 and eight (36.4%) at week 26, while PGA 0/1 (completely/almost clear) was achieved by five (22.7%) and 12 patients (54.5%) at week 6 and 26, respectively. Mean changes from baseline in pain numeric rating scale (−1.8 at week 6 and −2.5 at week 26) and the Dermatology Life Quality Index (−3.1 at week 6 and −3.6 at week 26) improved over time. Adverse events were reported by 18 patients, most commonly infections (*n* = 11), and serious adverse events by four. These results suggest that adalimumab is effective and generally well tolerated in Japanese patients with active ulcers of pyoderma gangrenosum.

## Introduction

Pyoderma gangrenosum (PG) is a rare, chronic, inflammatory disease characterized by deep and painful skin ulcers and is estimated to affect 3–10/million people per year.^1^, ^2^ The pathogenesis of PG is still poorly understood. Abnormalities in inflammatory cytokines and neutrophils and a genetic background have been shown to play a role in the pathophysiology.^3^, ^4^ In approximately half of cases, PG is associated with underlying systemic disease such as rheumatoid arthritis, inflammatory bowel disease or lymphoproliferative disorder.^1^, ^5^, ^6^ Diagnosis is often based on exclusion of skin neoplasms, infection, stasis ulcer and other inflammatory conditions.^2^, ^3^ For a definitive diagnosis of PG, skin biopsy is essential to exclude other dermatoses.^2^

Pyoderma gangrenosum is clinically classified into four types: ulcerative (classic), bullous, pustular and vegetative.^1^, ^7^ Of these, the ulcerative type is most common and characterized by rapidly enlarging deep central ulceration with undermined borders. Initially, a small sterile follicular pustule arises, rapidly forms an abscess, ulcerates and spreads outward. The ulcerations are surrounded by raised edematous borders, of which the surface is covered with necrotic tissue.^1^, ^7^ Most commonly, PG occurs in the lower legs, although other sites may also be impacted, such as the face, trunk and genital regions. Although the clinical manifestations of PG are variable, the subtypes of PG share a similar pathophysiological/histological course.

Although no treatment has currently been approved for PG, systemic corticosteroids are commonly prescribed as the first‐line therapy.^3^, ^6^ Oral cyclosporin has similar efficacy to oral corticosteroid as demonstrated in the randomized controlled STOP GAP trial.^8^ Systemic immunosuppressive and immunomodulatory drugs, including thalidomide, tacrolimus, dapsone and azathioprine, and topical immunotherapies, including corticosteroids, tacrolimus and dapsone, have also been used.^3^, ^6^ Tumor necrosis factor (TNF) inhibitors, including infliximab and adalimumab, have been reported to be effective when administrated to patients with PG ulcers in case reports and one small randomized study.^9^, ^10^, ^11^, ^12^, ^13^, ^14^ Because alternative medication is required for refractory PG, this open‐label study was conducted to evaluate the efficacy, safety and pharmacokinetics of adalimumab 40 mg every week for 26 weeks in Japanese patients with active ulcers of PG.

## Methods

### Study design and participants

**Figure 1:**
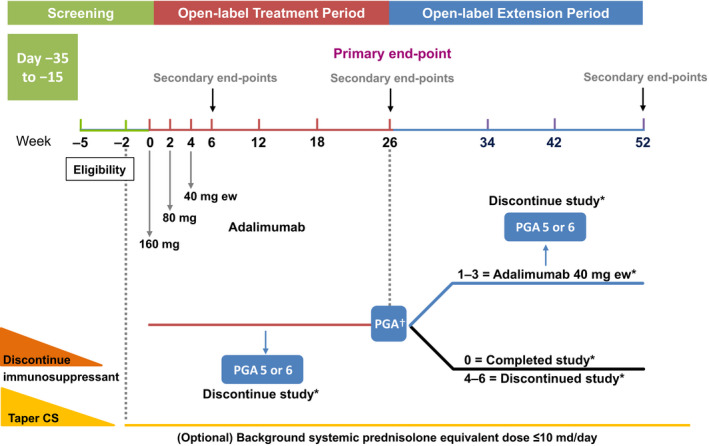
Study design. *Follow‐up: patients were contacted approximately 70 days following study drug discontinuation for an assessment of any new or ongoing adverse event (AE). ^†^Patients who reached improvement of ulcers with a Physician Global Assessment (PGA) score of 1–3 at week 26 could enter the extension period to receive adalimumab 40 mg every week until week 52. Patients who achieved healing of all ulcers (a PGA score of 0) at week 26 were considered to have completed the study, and patients who had a PGA score of 4–6 at week 26 were discontinued from the study. CS, corticosteroid; ew, every week.

This phase 3, open‐label, single‐arm, multicenter study enrolled patients at 15 study sites in Japan. The study included a screening period (of up to 5 weeks), a 26‐week treatment period and a 26‐week extension period (ongoing; Fig. 1). Patients with active PG ulcers were enrolled to receive adalimumab 160 mg s.c. at week 0, followed by 80 mg at week 2 and 40 mg every week starting at week 4. Patients who reached improvement of ulcers with a Physician’s Global Assessment (PGA) score of 1–3 at week 26 could enter the extension period to receive adalimumab 40 mg every week until week 52. Patients who achieved healing of all ulcers (a PGA score of 0) at week 26 were considered to have completed the study, and patients who had a PGA score of 4–6 at week 26 were discontinued from the study. Interim results from the first 26 weeks are reported here.

Adult patients (≥18 years) with a diagnosis of active ulcerative (classic) PG (including peristomal) made by the investigator and an inadequate response to or not a suitable candidate for topical PG therapy were eligible. Patients had to have at least one active and measurable ulcer with a distinct margin surrounded by epithelialized skin. The target PG ulcer criteria were 3 cm or more to less than 10 cm in its longest distance if it was a non‐peristomal ulcer, or 1 cm or more to less than 10 cm for a peristomal ulcer (excluding the stoma) at week −2. If the target PG ulcer size changed from week −2 to baseline, the size at week −2 determined the eligibility of the patient. Patients with pustular, bullous/atypical or vegetative variants of PG and those with clinical evidence of non‐PG‐related ulceration, vasculitis, thrombosis‐prone conditions, monoclonal gammopathy or active systemic viral infection were ineligible for enrollment. Patients with evidence of dysplasia or history of malignancy (including lymphoma and leukemia) other than a successfully treated non‐metastatic cutaneous squamous cell carcinoma, basal cell carcinoma or localized carcinoma *in situ* of the cervix were also ineligible. Patients with prior exposure to adalimumab or those who were receiving a biologic agent or systemic treatments for PG or who discontinued biologics or systemic treatments (e.g. cyclosporin, mycophenolic acid, azathioprine, diaphenylsulfone/dapsone or i.v. immunoglobulin) within five half‐lives of each drug prior to week −2 were excluded. Patients receiving azathioprine/6‐mercaptopurine, sulfasalazine, salazosulfapyridine, mesalazine, methotrexate or leflunomide for other concurrent indications (e.g. inflammatory bowel disease or rheumatoid arthritis) had to have been on these medications for at least 3 months prior to week −2 and/or been on a stable dose of these drugs for at least 4 weeks prior to week −2. Patients receiving an oral corticosteroid who tapered down to a prednisolone‐equivalent dose of 10 mg/day or less by the week −2 visit were eligible; if systemic corticosteroids were used for treating ulcer(s) at screening, patients underwent an oral corticosteroid tapering to a daily prednisolone‐equivalent dose of 10 mg or less by week −2 visit. If patients were unable to taper these drugs, they were allowed to return for a re‐screen. Patients receiving topical treatment for PG within 14 days prior to baseline visit were also excluded.

This study was conducted in accordance with the protocol, International Conference on Harmonisation guidelines, applicable regulations and the ethical principles of the Declaration of Helsinki. All patients reviewed and signed an informed consent form before any study procedures were undertaken. An independent ethics committee or institutional review board at each study site approved the study protocol, informed consent form and other study‐related documents.

### Efficacy and safety end‐points

The primary efficacy end‐point was the proportion of patients who achieved PG area reduction (PGAR) 100 (defined as complete skin re‐epithelialization) for the target ulcer at week 26 assessed by photography‐based digital measurements (digital planimetry). The key secondary end‐points related to the target PG ulcer included the proportion of patients achieving PGAR 100 at any time point through week 26, mean time to healing (defined as PGAR 100) through week 26, mean time to relapse (PGAR of <100) among patients who had achieved PGAR 100 prior to week 26, velocities of healing between week 0 and 6 and between week 6 and 26, percentage change from baseline in ulcer area (using digital planimetry), and proportion of patients with an Investigator Inflammation Assessment (IIA) score of 0 for both erythema and border elevation at week 6 and 26. In addition, the following key secondary end‐points were assessed to evaluate global improvement in patients (all PG ulcers, including the target PG ulcer were assessed): proportion of patients achieving PGA 0 or 0/1, change from baseline in pain as measured by numeric rating scale (NRS) and change from baseline in Dermatology Life Quality Index (DLQI) at week 6 and 26 as well as mean time to occurrence of new PG ulcers through week 26.

Safety monitoring, including collection of adverse events (AE), laboratory data, physical examinations and vital signs, was conducted throughout the study and recorded at designated study visits. All AE, serious AE, AE leading to discontinuation and prespecified AE of special interest were collected up to approximately 70 days after the last dose of study drug. The number and percentage of patients experiencing AE were tabulated using the Medical Dictionary for Regulatory Activities version 22.0 (MedDRA, McLean, VA, USA) system organ class and preferred term. A listing of all patients with any laboratory determination meeting Common Terminology Criteria for Adverse Events version 4.0 (National Institutes of Health, Bethesda, MD, USA) of grade 2 or higher was prepared.

### Statistical analyses

Efficacy was assessed in the full analysis set (FAS), which included all patients who received at least one dose of study drug and had at least one post‐treatment efficacy assessment. Safety was assessed in the safety population, which was defined as all patients who received at least one dose of study drug. A subgroup analysis in patients with baseline systemic corticosteroid use was also done.

Missing data were imputed using non‐responder imputation (NRI) and last observation carried forward in the FAS population; observed case analysis was also done. For the primary end‐point, a one‐sample χ^2^‐test was used with a threshold level of 20%. For secondary end‐points, the frequencies, percentages and 95% confidence interval (CI) were reported for binary end‐points, and the mean, standard deviation, median and range, and 95% CI were reported for continuous end‐points. The analyses were performed using SAS software (SAS Institute, Cary, NC, USA).

The study was designed to enroll approximately 20 patients due to the limited number of patients with PG in Japan. In the STOP GAP trial, the response rates of healing (defined as the condition where dressings were no longer required) by week 26 were 47% in both the oral corticosteroid and oral cyclosporin groups. Assuming the threshold response rate of 20% at week 26 as a clinically meaningful success of treatment and the expected clinical response rate of 47%, a sample size of 20 patients would have 80% power to detect the difference of 27% in the primary end‐point and using one‐sample χ^2^‐test at one‐sided 2.5% significance level.

## Results

**Figure 2:**
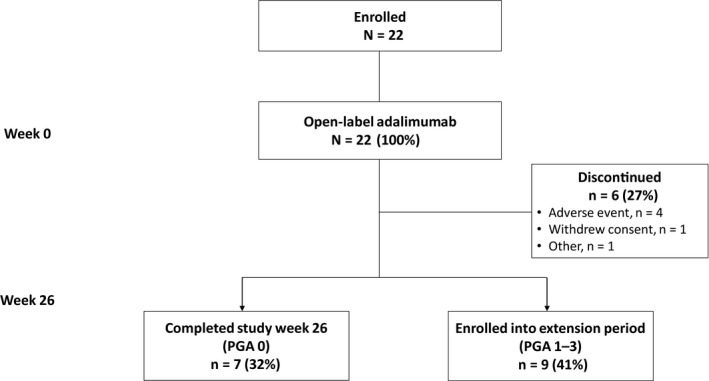
Patient disposition. PGA, Physician Global Assessment.

A total of 22 Japanese patients were enrolled and received open‐label adalimumab (Fig. 2). Of these, seven patients (32%) who achieved a PGA score of 0 completed the study at week 26 of the treatment period, and nine patients (41%) with PGA scores of 1–3 continued into the 26‐week extension period. Six patients (27%) discontinued the study drug during the 26‐week treatment period; the most frequently reported primary reason for discontinuation of the study drug was an AE (Fig. 2).

The mean age was 56.4 years (range, 21–83) and 55% of patients were female (Table 1). Target PG ulcer mean area was 33.3 cm^2^ (digital planimetry) at baseline, and the target PG ulcer was most commonly located on a leg (18/22 [82%]; other locations included [*n* = 1 for each] arm, buttocks, head and foot). The mean duration of PG was 3.3 years. Baseline comorbidities included hypertension (45% of patients), hyperlipidemia (27%), hyperuricemia (23%), osteoporosis and ulcerative colitis (18% each), and rheumatoid arthritis (14%); one patient presented with peristomal dermatitis. Mean pain NRS was 4.6 and DLQI was 9.3. Sixteen patients (73%) had concomitant corticosteroid use at baseline, including 13 (59%) who used corticosteroids for PG (Table 1). One patient had previously received etanercept for PG.

**Table 1:** Baseline demographics and disease characteristics

	Adalimumab *n* = 22
Female, *n* (%)	12 (55)
Age, years	
Mean (SD)	56.4 (18.6)
Median (range)	60.5 (21.0–83.0)
Weight, kg	67.5 (21.7)
Disease duration, years	3.3 (5.3)
Comorbidities, *n* (%)	
Hypertension	10 (45)
Hyperlipidemia	6 (27)
Hyperuricemia	5 (23)
Osteoporosis	4 (18)
Ulcerative colitis	4 (18)
Rheumatoid arthritis	3 (14)
Target PG ulcer area (digital planimetry), cm^2^	33.3 (25.2)
IIA, moderate to very severe, *n* (%)	
Erythema	16 (73)
Border elevation	15 (68)
Pain (NRS)	4.6 (3)
DLQI	9.3 (7)
Baseline corticosteroid use, *n* (%)	16 (73)
Corticosteroid use for PG	13 (59)

Mean (standard deviation [SD]) data reported unless noted otherwise. DLQI, Dermatology Life Quality Index; IIA, Investigator Inflammation Assessment; NRS, numeric rating scale; PG, pyoderma gangrenosum.

### Efficacy end‐points

**Figure 3:**
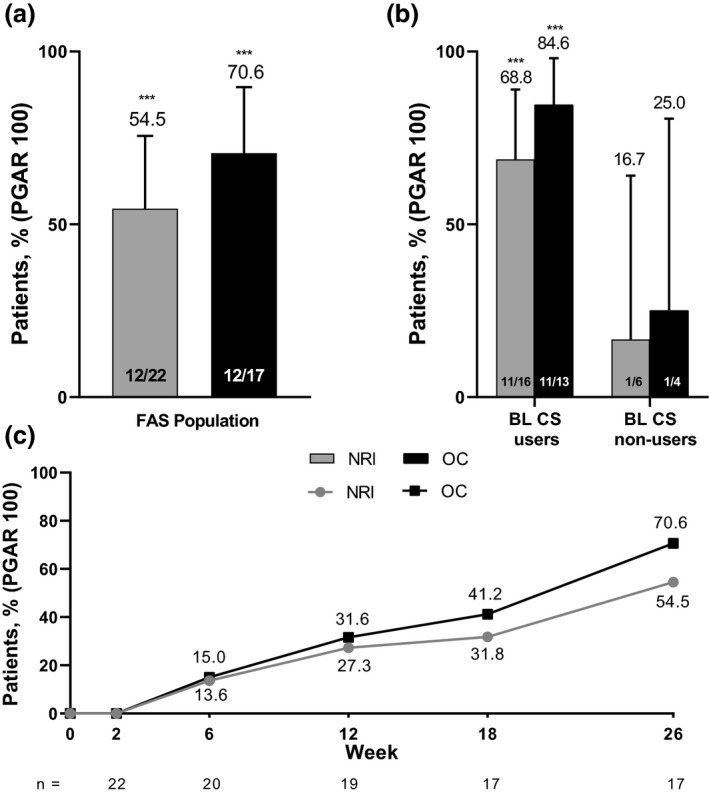
(a) Primary end‐point pyoderma gangrenosum area reduction 100 (PGAR 100, defined as complete skin re‐epithelialization) at week 26 full analysis set (FAS) population, (b) subgroup analysis of PGAR 100 among patients with baseline systemic corticosteroid use and (c) PGAR 100 over time. BL, baseline; CS, corticosteroid; NRI, non‐responder imputation; OC, observed case. ***P < 0.001.

At week 26, 12 patients achieved the primary end‐point of PGAR 100 for the target ulcer by digital planimetry (54.5% [95% CI, 32.2%–75.6%], *P* < 0.001, NRI analysis). Results were similar in the observed case analysis in which 12 of 17 patients (70.6% [95% CI, 44.0%–89.7%], *P* < 0.001) achieved PGAR 100 at week 26 (Fig. 3a). PGAR 100 response was observed as early as week 6 (13.6%, NRI analysis) and continued to increase over time (Fig. 3c). In the subgroup analysis, 11 of 16 patients (68.8% [95% CI, 41.3%–89.0%], *P* < 0.001) taking baseline systemic corticosteroids achieved PGAR 100 for the target ulcer at week 26 (Fig. 3b).

**Figure 4:**
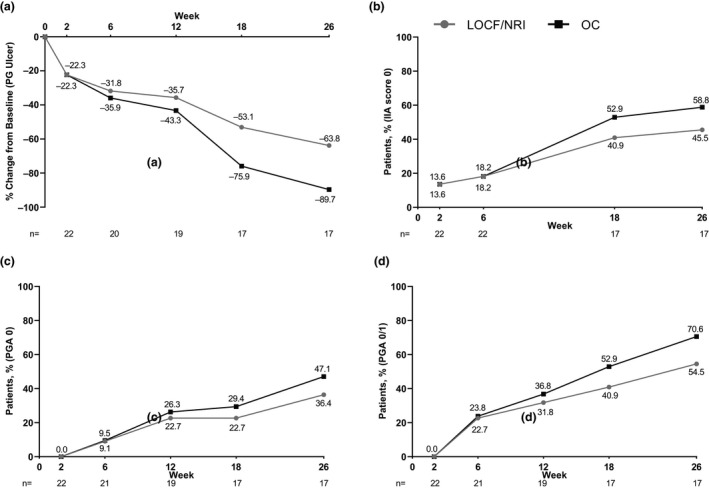
(a) Percentage change from baseline in target pyoderma gangrenosum (PG) ulcer area, and percentage of patients achieving (b) IIA score 0, (c) Physician Global Assessment (PGA) 0 and (d) PGA 0/1 over time. IIA, Investigator Inflammation Assessment; LOCF, last observation carried forward (panel a); NRI, non‐responder imputation (panels b–d); NRS, numeric rating scale; OC, observed case.

The mean percent change from baseline in the target ulcer area by digital planimetry improved over time, reaching −31.8% at week 6 and −63.8% at week 26 (Fig. 4a). The proportion of patients achieving an IIA score of 0 for the target ulcer also increased throughout the 26 weeks (from 18.2% at week 6 to 45.5% at week 26; Fig. 4b). The mean time to healing of the target ulcer was 114.9 days. No patients who achieved PGAR 100 for the target ulcer before week 26 experienced a relapse within the 26 weeks. The mean velocity of healing was −0.21 cm^2^/day between baseline and week 6, and −0.11 cm^2^/day between baseline and week 26.

**Figure 5:**
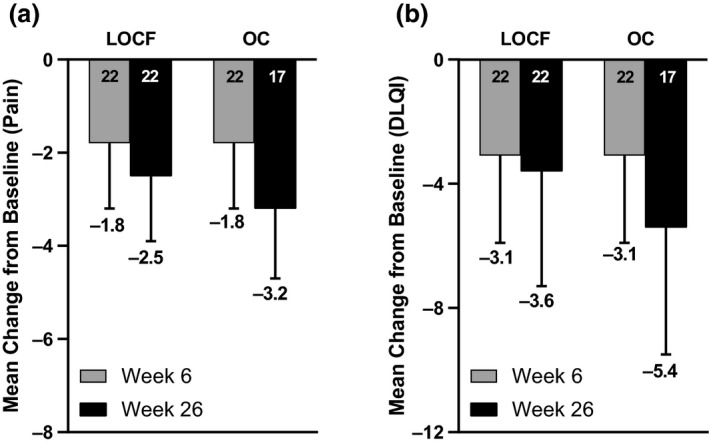
Mean change from baseline in (a) patient pain and (b) Dermatology Life Quality Index (DLQI) at week 6 and 26. LOCF, last observation carried forward; OC, observed case.

Two patients (9.1%) achieved PGA 0 at week 6, which increased to eight patients (36.4%) by week 26 (Fig. 4c). PGA 0/1 was achieved by five (22.7%) and 12 patients (54.5%) at week 6 and 26, respectively (Fig. 4d). The mean change from baseline in NRS pain scale improved over time, increasing from −1.8 at week 6 to −2.5 at week 26 (Fig. 5a). Eleven (50%) of the 22 patients used analgesics at baseline. This number decreased to eight patients (36%; *n* = 22) by week 6, and continued to decrease to two patients (12%, *n* = 17) by week 26. For DLQI, the mean change from baseline was −3.1 at week 6 and −3.6 at week 26 (Fig. 5b). Seven patients reported new ulcers during the 26‐week treatment period; the mean time to occurrence of new ulcers was 22.1 days.

### Safety

Overall, 18 patients (82%) experienced at least one treatment‐emergent AE during the 26‐week treatment period, and four patients (18%) experienced an AE leading to study drug discontinuation (Table 2). Four patients (18%) experienced a serious AE (one event each of anemia, bacterial arthritis, cataract and pain due to PG), one of which led to discontinuation of the study drug (bacterial arthritis). Most AE were mild or moderate in severity; two patients (9%) experienced three AE assessed as severe. In the first patient, these included severe pain, assessed by the investigator as not related to the study drug which was not interrupted, and severe PG aggravated, assessed by the investigator as having reasonable possibility of being related to the study drug which was discontinued. The second patient experienced severe bacterial arthritis (event of pyogenic arthritis of the right knee), assessed by the investigator as having a reasonable possibility of being related to the study drug, which was discontinued. The patient was an 84‐year‐old woman with a medical history of osteoporosis, osteoarthritis and right knee replacement.

**Table 2:** Treatment‐emergent adverse events

	Adalimumab *n* = 22
Treatment‐emergent AE *n* (%)	
Any AE	18 (82)
Severe AE	2 (9)^†^, ^‡^
Serious AE	4 (18)^†^, ^‡^
AE leading to discontinuation of study drug	4 (18)^†^, ^‡^
Serious AE leading to discontinuation of study drug	1 (5)^†^, ^‡^
AE possibly related to study drug	9 (41)
Serious AE possibly related to study drug	2 (9)
AE of special interest	
Infection	11 (50)
Serious infection	1 (5)^†^, ^‡^
Opportunistic infections	0
Oral candidiasis	0
Tuberculosis	0
Malignancy	0
Allergic reaction (including angioedema/anaphylaxis)	2 (9)^†^, ^‡^
Injection‐site reaction	1 (5)
Deaths	0

^†^ Severe pain and severe pyoderma gangrenosum (PG) aggravated in one patient and severe bacterial arthritis in one patient.

^‡^ Anemia, bacterial arthritis, cataract and pain due to PG.

^§^ One adverse event (AE) each of bacterial arthritis, cellulitis, skin bacterial infection, drug eruption, PG (PG aggravated) and toxic skin eruption.

^¶^ Bacterial arthritis.

^††^ Drug eruption and urticaria.

The most frequently reported AE were anemia, Cushingoid, eczema, nasopharyngitis and PG (each in three patients [14%]). Nine patients (41%) experienced AE assessed by the investigator as having at least a reasonable possibility of being related to the study drug; four (18%) were classified as mild, three (14%) as moderate and two (9%) as severe (PG and bacterial arthritis).

No events of opportunistic infection, tuberculosis (active or latent), progressive multifocal leukoencephalopathy, malignancy, intestinal perforation, and cardiopulmonary‐ or liver‐related AE were reported. One patient died during the screening period due to acute ascending aortic dissection; no other deaths were reported in the study.

No clinically meaningful changes in mean hematology values were observed. A few patients had potentially clinically significant (grade ≥3) hematology values for low hemoglobin (1/21 [5%] grade 3) and lymphocytes (1/21 [5%] grade 3); neither patient discontinued the study due to these elevated laboratory values. Similarly, no clinically meaningful changes in mean clinical chemistry values were observed. A few patients had potentially clinically significant (grade ≥3) clinical chemistry values for urate (2/21 [10%] grade 4), cholesterol (1/21 [5%] grade 4) and triglycerides (1/21 [5%] grade 3 and 1/21 [5%] grade 4); none discontinued the study due to these elevated laboratory values. No patient met the criteria for Hy’s law (i.e. alanine aminotransferase or aspartate aminotransferase elevation >3 × upper limit of normal [ULN], concurrent bilirubin elevation >2 × ULN and symptomology), and no patient experienced an elevated laboratory value that caused them to discontinue the study drug.

## Discussion

This study assessed the efficacy and safety of adalimumab for the treatment of active ulcers due to PG. The primary efficacy end‐point, PGAR 100 of the target ulcer, was achieved by the majority of patients at week 26 (55%, *P* < 0.001). In a subgroup analysis by baseline systemic corticosteroid use, 69% of patients achieved PGAR 100 at week 26 (*P* < 0.001). The target PG ulcer area steadily improved over time, with 64% reduction at week 26, indicating a continuous reduction in the ulcer with continuous adalimumab treatment. The mean time to healing of the target ulcer was 114.9 days (16 weeks) and no relapses of target ulcer with PGAR 100 were observed during the 26 weeks. The proportion of patients achieving PGA 0/1 showed improvement and steadily increased over time, reaching 55% at week 26; similarly, the proportion of patients achieving PGA 0 reached 36% by week 26. The mean change from baseline in pain NRS also improved over time, and the mean improvement from baseline in DLQI that was achieved at week 6 was maintained at that level to week 26. The other secondary end‐point analyses were consistent with the primary analysis, demonstrating that adalimumab was effective in Japanese patients with active ulcers due to PG.

Because no treatment is currently approved for the treatment of PG, TNF inhibitors, including adalimumab, are often used off‐label. Only a few, small (10–52 patients) retrospective or randomized controlled TNF inhibitor studies in PG have been conducted to date, all of which demonstrated treatment response.^9^, ^15^, ^16^ Furthermore, the effectiveness of adalimumab for the treatment of PG has previously been reported in numerous case studies.^10^, ^11^, ^13^, ^14^, ^17^ Healing of PG ulcers of up to 450 cm^2^ was noted after 2–16 months of treatment. Based on the previous reports, an adalimumab dosing regimen of 160 mg initially, followed by 80 mg at week 2 and 40 mg every week thereafter, was chosen for this study. After 26 weeks of adalimumab treatment, seven (32%) patients achieved healing of all ulcers (a PGA score of 0) and completed the study. The remaining nine (41%) patients with PGA score of 1–3 at week 26 were enrolled in the ongoing extension study, which will provide further long‐term data on the efficacy and safety of adalimumab in this patient population.

The safety profile of adalimumab was consistent with the known safety profile of adalimumab, and no new or unexpected AE were identified over previous studies. The most frequently reported AE were anemia, Cushingoid, eczema, nasopharyngitis and PG. One death was reported during the screening period; no other deaths or cases of opportunistic infection, tuberculosis, progressive multifocal leukoencephalopathy, malignancy, intestinal perforation or cardiopulmonary‐ or liver‐related AE were reported.

The strengths of this study include a well‐balanced population of Japanese patients with confirmed ulcerative PG, with a wide age range of the patients of 21–83 years and treatment for up to 1 year. This study was limited by the open‐label, single treatment design that could potentially contribute to data bias because no double‐blind control group was included; nevertheless, consistent efficacy was demonstrated for primary and secondary end‐points. In addition, the small number of patients due to the low prevalence of PG is a limitation of this study; however, the primary end‐point met the assumptions used to determine the sample size. Finally, patients receiving concomitant low‐dose corticosteroids were allowed to enroll in the study; however, this is common in clinical practice and consistent improvements with adalimumab were observed in patients regardless of concomitant corticosteroid use.

In conclusion, 26‐week open‐label treatment with adalimumab 40 mg every week demonstrated complete skin re‐epithelialization of PG ulcers in the majority of Japanese patients with active PG ulcers. Adalimumab therapy was generally well tolerated, and reported AE were consistent with the known safety profile of adalimumab. These results suggest that adalimumab is a beneficial therapy option for patients with active ulcers due to PG.

## Conflict of Interest

K. Yamasaki is an advisor for this study but has no conflicts of interest for this study based on Japanese Dermatology Association criteria. K. Yamanaka has received research grants, speaker’s fees and chair’s fees from AbbVie. Y. Z., S. I., K. T. and K. S. are full‐time salaried employees of AbbVie. T. Y. has no conflicts of interest to declare.

## Data Availability

AbbVie is committed to responsible data sharing regarding the clinical trials we sponsor. This includes access to anonymized, individual and trial‐level data (analysis datasets), as well as other information (e.g. protocols and Clinical Study Reports), as long as the trials are not part of an ongoing or planned regulatory submission. This includes requests for clinical trial data for unlicensed products and indications. This clinical trial data can be requested by any qualified researchers who engage in rigorous, independent scientific research, and will be provided following review and approval of a research proposal and Statistical Analysis Plan (SAP) and execution of a Data Sharing Agreement (DSA). Data requests can be submitted at any time and the data will be accessible for 12 months, with possible extensions considered. For more information on the process, or to submit a request, visit the following link: https://www.abbvie.com/our‐science/clinical‐trials/clinical‐trials‐data‐and‐information‐sharing/data‐and‐information‐sharing‐with‐qualified‐researchers.html.
